# A Robust DOA Estimator Based on Compressive Sensing for Coprime Array in the Presence of Miscalibrated Sensors

**DOI:** 10.3390/s19163538

**Published:** 2019-08-13

**Authors:** Jiaxun Kou, Ming Li, Chunlan Jiang

**Affiliations:** State Key Laboratory of Explosion Science and Technology, Beijing Institute of Technology, Beijing 100081, China

**Keywords:** robust direction-of-arrival (DOA), coprime array, compressive sensing (CS), calibration error, outlier, maximum correntropy criterion (MCC)

## Abstract

Coprime array with M+N sensors can achieve an increased degrees-of-freedom (DOF) of O(MN) for direction-of-arrival (DOA) estimation. Utilizing the compressive sensing (CS)-based DOA estimation methods, the increased DOF offered by the coprime array can be fully exploited. However, when some sensors in the array are miscalibrated, these DOA estimation methods suffer from degraded performance or even failed operation. Besides, the key to the success of CS-based DOA estimation is that every target falls on the predefined grid. Thus, a coarse grid may cause the mismatch problem, whereas a fine grid requires great computational cost. In this paper, a robust CS-based DOA estimation algorithm is proposed for coprime array with miscalibrated sensors. In the proposed algorithm, signals received by the miscalibrated sensors are viewed as outliers, and correntropy is introduced as the similarity measurement to distinguish these outliers. Incorporated with maximum correntropy criterion (MCC), an iterative sparse reconstruction-based algorithm is then developed to give the DOA estimation while mitigating the influence of the outliers. A multiresolution grid refinement strategy is also incorporated to reconcile the contradiction between computational cost and the mismatch problem. The numerical simulation results verify the effectiveness and robustness of the proposed method.

## 1. Introduction

Direction-of-arrival (DOA) estimation is a vital technology in the field of array signal processing that has been widely applied in radar, sonar, acoustic, navigation, and wireless communication [1,2,3]. Traditionally, uniform linear array (ULA) has been the most commonly used array configuration because of its simplicity for application and well-developed techniques. The main disadvantage of using ULA is that the achievable number of degrees-of-freedom (DOFs) of a ULA consisting of *M* sensors is limited to M−1. To break through this limitation, coprime arrays were proposed in Ref. [4], which provide a larger array aperture than the ULA with the same number of sensors and can resolve up to O(MN) sources with M+N−1 sensors.

In order to take advantage of the DOF superiority offered by the coprime array, several techniques have been developed for DOA estimation, including spatial smooth [5], array interpolation [6] and compressive sensing [7]. Among them, compressive sensing (CS) is one of the most promising signal-processing techniques [8], which can accurately recover signal at a sub-Nyquist sampling rate. The CS-based DOA estimation algorithms can be divided into three categories: greedy algorithms, such as orthogonal matching pursuit (OMP) [9]; convex relaxation algorithms, such as least absolute shrinkage and selection operator (LASSO) [10]; and Bayesian CS, such as sparse Bayesian learning (SBL). In Ref. [7], a sparse reconstruction method based on LASSO was proposed for coprime arrays, which fully utilizes the entire virtual array aperture. Combining the compressive measurement method with sparse array, Guo et al. proposed two categories of methods in Ref. [11] to significantly reduce the system complexity.

However, excellent performance of the CS-based DOA estimation methods is critically dependent on the precondition that all the sensors in the array are properly calibrated. Otherwise, sensor miscalibration will deteriorate the performance of these methods, and even lead to operation failure. Therefore, it is clear that array calibration is important to ensure robust DOA estimation in practice. In recent decades, two branches of the research have been conducted to address this miscalibration problem [12]. The first branch is called active calibration that requires auxiliary sensors or co-operation signal sources with known DOAs. In Ref. [13], the good calibrating performance is acquired by placing the known sources in space and storing the array manifold of the uncalibrated array excited by these sources. When the number of calibrated sensors is more than the number of signal sources and the number of signal sources is more than one, the partly calibrated method proposed in Ref. [14] also has excellent performance. Nevertheless, the requirements for using active calibration methods are often difficult to meet, making them difficult to implement in practice. Unlike active calibration methods, the other branch, referred to as self-calibration methods, does not have the need for assistant sources or sensors. Under the assumption that all the sensor miscalibrations can be modeled as unknown but deterministic gain and phase error parameters, several self-calibration methods have been proposed on the basis of CS methods, which jointly estimate these error parameters with DOAs. Just like the classification of CS methods, these self-calibration methods can be also divided into the same three categories. In Ref. [15], a method was proposed under the framework of OMP, which treats the gain/phase uncertainties as an additive error matrix by transferring the array signal model with gain/phase uncertainties into an error-in-variables (EIV) model. A total least squares (TLS) problem was then formulated and the simultaneous orthogonal matching pursuit (SOMP) algorithm was introduced to solve this problem. Although the authors claim that their method can be applied to the sparse array, the enhanced DOF offered by the sparse array is not exploited throughout this paper. Besides, since this method discretizes the signal space into grids and assumes that all targets fall on the predefined grid, it may have a grid mismatch problem. To address the mismatch problem, Camlica et al. proposed another OMP-based method in Ref. [16] for off-grid signals. In this method, a non-linear cost function is approximated by its first order Taylor series for the sake of simplicity. Thus, this method works well only when the perturbations are not severe. These two OMP-based methods have some common shortcomings: first, the number of sources must be known a priori; second, they can only obtain suboptimal solutions through non-convex models. Meanwhile, some other self-calibration methods are developed under the framework of LASSO. In Ref. [17], the calibration problem is transformed into a sparse matrix completion problem. This problem is non-convex; it is then decomposed into two convex subproblems so that it can be efficiently solved. However, this method adopts the same linearization strategy as the method in Ref. [16], so it cannot be applied to the cases of serious disturbance either. In Ref. [18], a convex optimization approach was proposed to blindly calibrate the sensor array. Unfortunately, the applied range of this approach is very limited because it can only work under the noise-free condition. The methods proposed in Refs. [19] and [20] exploit the orthogonality property between the signal subspace and noise subspace, so they cannot be implied for the coprime array with the expectation to find more sources than sensors. Under the framework of SBL, Lu et al. proposed a method for nested array calibration [21], which employs the EM algorithm to solve a non-convex optimization problem and thus jointly estimates the DOAs with the error parameters. This method is able to exploit the enhanced DOF offered by the nested array; however, it is computationally intensive and its convergence is not guaranteed. In Ref. [22], a robust CS-based DOA estimation method was proposed under the assumption that few sensors in the array are miscalibrated. Unlike the methods that explicitly estimate the error parameters, this method treats the miscalibrated sensor observations as outliers and a weighting factor is adaptively optimized and applied to mitigate the effect of the outliers.

Inspired by Ref. [22], in this paper, a robust CS-based DOA estimation method is proposed for coprime array in the presence of miscalibrated sensors, which can be used to find more sources than sensors. Similar to Ref. [22], it is assumed that the sensor miscalibration occurs randomly in the array, where both the number and the positions of the miscalibrated sensors are not known a priori. Signals received by these miscalibrated sensors are viewed as outliers, and the correntropy is employed as the similarity measurement to distinguish them. The sparse signal recovery is formulated as an optimization problem, in which the maximum correntropy criterion (MCC) is employed as the constraint condition to suppress the influence of the sensor miscalibration. Exploiting the property of conjugate function, an iterative algorithm is developed under the convex relaxation framework to effectively solve the optimization problem. Furthermore, a grid refinement strategy is employed to alleviate the grid mismatch problem, with which the grids are adaptively refined at each iteration. The DOA estimation is thus acquired after the convergence of this iterative algorithm. The proposed method fully uses the virtual aperture constructed by the coprime array structure, and achieves accurate DOA estimation with enhanced DOF even when array sensors are severely perturbed. Numerical simulations are conducted to verify the effectiveness and robustness of the proposed method.

The rest of this paper is organized as follows. In Section 2, the signal model used through this paper is set up, and then the effect of miscalibrated sensors is discussed. The theories of CS-based DOA estimation method and MCC are reviewed in Section 3. Based on the theories reviewed in Section 3, a robust DOA estimation algorithm for coprime array is proposed in Section 4 with a multiresolution grid refinement strategy, where the Cramér-Rao Bound (CRB) of DOA estimation is also given. The simulation results are shown in Section 5. Finally, the conclusions are made in Section 6.

Notations: The lower-case boldface characters, upper-case boldface characters, and upper-case characters in blackboard boldface are used to denote vectors, matrices, and sets respectively throughout this paper. ${\mathbb{C}}^{M\times N}$ denotes a complex matrix or vector (when *N* = 1). The superscripts ${\left(\cdot \right)}^{T}$, ${\left(\cdot \right)}^{H}$ and ${\left(\cdot \right)}^{*}$ denote the transpose, conjugate transpose, and complex conjugation, respectively. ${\left(\cdot \right)}^{-1}$ and tr(⋅) respectively denote the inverse and the trace of a matrix. ${\left[A\right]}_{i,j}$ indicates the (*i*, *j*)-th entry of A. The square bracket notation of a vector ${\left[x_{S}\right]}_{i}$ represents the *i*-th component of $x_{S}$. For n∈S, the triangular bracket notation ${\langle x_{S}\rangle }_{n}$ denotes the signal value at the support location *n*, where the detailed definition is given in Ref. [23]. |⋅| denotes the cardinality of a set. The notation E[⋅] denotes the statistical expectation. vec(⋅) stands for the vectorization operator that sequentially stacks each column of a matrix, and diag(⋅) represents a diagonal matrix with the corresponding elements on its diagonal. The symbols ⊙ and ⊗ represent the Khatri-Rao product and Kronecker product respectively. I denotes the identity matrix with an appropriate dimension. Finally, the symbol *j* represents imaginary unit $\sqrt{-1}$.

## 2. Problem Formulation

### 2.1. Signal Model of Coprime Array

**Figure 1:**
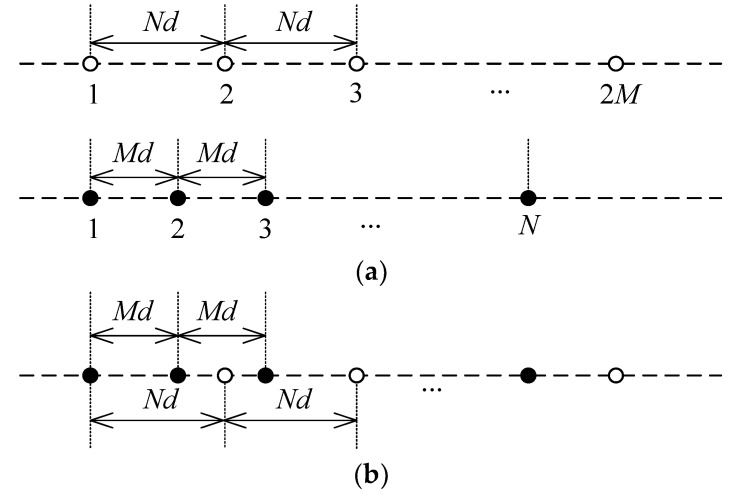
Illustration of an extended coprime array. (**a**) A coprime pair of sparse ULAs; (**b**) Coprime array configuration, which is a combination of the two sparse ULAs above.

An extended coprime array, as depicted in Figure 1, consists of a pair of sparsely-spaced ULAs. Denote *M* and *N* to be a pair of mutually coprimed integers, and without loss of generality, it is assumed that *M* < *N*. Then, the sparse ULA consisting of 2*M* sensors has an inter-element spacing of *N* units, whereas the other sparse ULA consisting of *N* sensors has an inter-element spacing of *M* units. Specifically, a total of 2M+N−1 sensors are located at

(1) S={Mnd|0≤n<N−1}≤{Nmd|0≤m≤2M−1},

where d=λ/2 denotes the unit inter-element spacing, and λ is the wavelength.

Assuming that *K* far-field uncorrelated narrowband sources impinges from directions $\theta ={\left[\theta_{1},\theta_{2},\ldots ,\theta_{K}\right]}^{T}$, the received signal vector of the coprime array at time *t* can be modeled as

(2) $$x(t)=\sum_{k=1}^{K}a\left(\theta_{k}\right)s_{k}(t)+n(t)=As(t)+n(t),$$

where $A=\left[a\left(\theta_{1}\right),a\left(\theta_{2}\right),\ldots ,a\left(\theta_{K}\right)\right]\in {\mathbb{C}}^{(2M+N-1)\times K}$ is the array steering matrix, $s(t)={\left[s_{1}(t),s_{2}(t),\ldots ,s_{K}(t)\right]}^{T}$ is the signal waveform vector, and $n(t)∼CN(0,\sigma_{n}^{2}I)$ denotes the independent and identical distributed (i.i.d) zero-mean addictive white Gaussian noise vector. Here, $\sigma_{n}^{2}$ represents noise power. The *k*-th column of **A** is the steering vector of the *k*-th source, expressed by

(3) $$a(\theta_{k})={\left[1,e^{-j\frac{2\pi }{\lambda }l_{2}\sin\left(\theta_{k}\right)},\ldots ,e^{-j\frac{2\pi }{\lambda }l_{2M+N-1}\sin\left(\theta_{k}\right)}\right]}^{T},$$

where $l_{i}\in S$, i=1,2,…,2M+N−1, denotes the position of the *i*-th sensor in the array with $l_{1}=0$.

Denoting the covariance matrix of the received signal as $R_{x}$, then it can be written as

(4) $$R_{x}=E\left[x(t)x^{H}(t)\right]=\sum_{k=1}^{K}p_{k}a\left(\theta_{k}\right)a^{H}\left(\theta_{k}\right)+\sigma_{n}^{2}I,$$

where $p_{k}$ is the power of *k*-th source. In practice, since the exact $R_{x}$ is unavailable, it is approximated by its maximum likelihood (ML) estimation, computed by

(5) $${\hat{R}}_{x}=\frac{1}{T}\sum_{t=1}^{T}x(t)x^{H}(t),$$

where *T* represents the number of snapshots.

### 2.2. Effect of Miscalbrated Sensors

Considering that a few sensors in the array are affected by unknown gain and phase distortions, the number of which is less than half of the number of sensors in the array, and they are known as miscalibrated sensors. Let M be a set of these miscalibrated sensors, then the cardinality of M is less than half of the number of sensors in the array, i.e., $\left|M\right|<\frac{1}{2}(2M+N-1)$. More specifically, if the *m*-th sensor is miscalibrated, the signals received by it can be modeled as

(6) $$x_{m}^{o}(t)=\sum_{k=1}^{K}\beta_{m}a_{m}(\theta_{k})s_{k}(t)+n_{m}(t),$$

where $\beta_{m}=\rho_{m}e^{j\varphi_{m}}$ is the unknown distortion parameter while $\rho_{m}$ and $\varphi_{m}$ stand for gain and phase distortion respectively; $a_{m}\left(\theta_{k}\right)$ denotes the *m*-th entry of $a(\theta_{k})$.

The signals received by the miscalibrated sensors are viewed as outliers. In such a manner, the received signal vector in Equation (2) becomes

(7) $$x^{o}(t)=\Gamma As(t)+n(t),$$

where Γ is a diagonal matrix whose *m*th entry is expressed as

(8) $$\Gamma_{m,m}=\beta_{m}=\left\{ \begin{array}{ll} \rho_{m}e^{j\varphi_{m}}, & m\in M\\ 1, & m\notin M\; \end{array}\right..$$

Γ being a diagonal matrix makes the calibration errors independent among different sensors. It is obvious that the actual steering matrix of the array in the presence of miscalibrated sensors becomes ΓA, which is apparently different from the original one, i.e., A. If the difference between these two steering matrixes is ignored in the DOA estimation process, it will lead to inaccuracy or even failed estimations. Therefore, the robust DOA estimation method is needed to combat the gain and phase distortions.

## 3. Related Works

### 3.1. Compressive Sensing-Based DOA Estimator

The CS-based methods estimate DOAs by exploiting the spatial sparsity in the direction of the sources. In the distortionless case, vectorizing the covariance matrix $R_{x}$ yields a ${\left(2M+N-1\right)}^{2}\times 1$ vector, expressed as

(9) $$y=\mathrm{vec}(R_{x})=\tilde{A}p+\sigma_{n}^{2}i,$$

where $\tilde{A}=A^{*}⊙A\in {\mathbb{C}}^{{\left(2M+N-1\right)}^{2}\times K}$ is the generalized steering matrix, $p={\left[p_{1},p_{2},\ldots ,p_{K}\right]}^{T}$, and i=vec(I).

To exploit the sparsity of DOAs, the spatial domain is usually discretized to create a dictionary that sparsely represents DOAs. The dictionary is built through grid sampling over the potential angular region (i.e., from $-90^{\circ }$ to $90^{\circ }$), and the grid points are denoted as ${\bar{\theta }}_{1},{\bar{\theta }}_{2},\ldots ,{\bar{\theta }}_{D}$. Here, *D* is the number of grid points, which is much larger than the number of sources, i.e., *D* >> *K*. It is noted that there are holes in the difference coarray constructed from a coprime array, i.e., the virtual sensors in the coarray are non-consecutive. Nevertheless, the CS-based methods do not need to consider the continuity issue of the coarray, and the dictionary can be directly utilized [24]. Therefore, the DOA estimation problem amounts to searching for the sparsest solution of vector $\bar{p}$ satisfying the following constraint [7]

(10) $$y=\bar{A}\bar{p}+\sigma_{n}^{2}i$$

where $\bar{A}=\left[\bar{a}\left({\bar{\theta }}_{1}\right),\ldots ,\bar{a}\left({\bar{\theta }}_{D}\right)\right]$ is the manifold dictionary matrix defined over *D* grid points, with $\bar{a}\left({\bar{\theta }}_{d}\right)=a^{*}\left({\bar{\theta }}_{d}\right)⊗a\left({\bar{\theta }}_{d}\right),\;d=1,\ldots ,D$. In this manner, the DOA estimation problem can be formulated as a constrained optimization problem, expressed by

(11) $$\underset{\bar{p}}{\min}\;{\left\|\bar{p}\right\|}_{0}\;s.t.\;{\left\|y-\bar{A}\bar{p}\right\|}_{2}^{2}\leq \varepsilon$$

where ${\left\|\cdot \right\|}_{0}$ is the $l_{0}$ norm of a vector, namely, the number of non-zero entries in a vector, ${\left\|\cdot \right\|}_{2}$ is the $l_{2}$ norm of a vector, and ε is a user-specific tolerance parameter. The $l_{0}$ norm is non-convex, which makes the problem in Equation (11) an NP-hard (Non-deterministic Polynomial) problem. One of the most important techniques to address this problem is the LASSO method, in which the $l_{0}$ norm is relaxed to $l_{1}$ norm, and the optimization problem in Equation (11) is represented in an unconstrained form, given by

(12) $$\underset{\bar{p}}{\min}\;\mu {\left\|\bar{p}\right\|}_{1}\;+\;{\left\|y-\bar{A}\bar{p}\right\|}_{2}^{2}$$

where μ is a regularization parameter that balances the sparsity and accuracy. Denoting the solution of Equation (12) as ${\bar{p}}^{*}$, then only *K* non-zero elements are expected in ${\bar{p}}^{*}$. The DOA is thus obtained by picking up the corresponding positions of these non-zero elements in ${\bar{p}}^{*}$.

### 3.2. MCC Theory

Correntropy is a generalized similarity measure between two arbitrary random variables *X* and *Y*, defined by [25]

(13) $$V_{\sigma }(X,Y)=E[\kappa_{\sigma }(X-Y)],$$

where

(14) $$\kappa_{\sigma }(X-Y)=\frac{1}{\sqrt{2\pi \sigma }}\exp\left(-\frac{{\left(X-Y\right)}^{2}}{2\sigma^{2}}\right)$$

is the Gaussian kernel function, and σ is the kernel size.

In practice, the joint probability density function (pdf) of *X* and *Y* is unknown, while only a finite number of data ${\left\{x_{i},y_{i}\right\}}_{i=1}^{N}$ are available. In this case, the correntropy can be approximated the by its sample version

(15) $${\hat{V}}_{N,\sigma }(X,Y)=\frac{1}{N}\sum_{i=1}^{N}\kappa_{\sigma }(x_{i}-y_{i}).$$

Conventionally, the minimum mean square error (MMSE) criterion is applied in the CS-based DOA estimation method as the constraint. Since mean square error (MSE) has the net effect of amplifying the contribution of samples that are far from the average value of error distribution, it makes MMSE criterion non-optimal when the error distribution has an outlier. As for correntropy, its value is primarily dictated by the kernel function along the x=y line. Based on this property of correntropy, the maximum correntropy criterion (MCC) is proposed, which is insensitive to the outliers [25]. Denoting E=Y−X as the fitting errors, and θ as a set of adjustable parameters, then MCC is defined as

(16) $$\begin{array}{l} \underset{\theta }{\max}\;E\left[\kappa_{\sigma }\left(E\right)\right]\\ \Leftrightarrow \underset{\theta }{\max}\;\int_{e}\kappa_{\sigma }\left(e\right)f_{E}(e)de \end{array}$$

Its sample version is defined as

(17) $$\underset{\theta }{\max}\;\frac{1}{N}\sum_{i=1}^{N}\kappa_{\sigma }\left(e_{i}\right).$$

## 4. Proposed Method

### 4.1. Robust DOA Estimator

As discussed in Section 2.2, when there exists some miscalibrated sensors in the coprime array, the actual steering matrix becomes ΓA. Therefore, the vectorization procedure of the covariance matrix in Equation (9) becomes

(18) $$y^{o}=\mathrm{vec}(R_{x}^{o})={\tilde{A}}^{o}p+\sigma_{n}^{2}i,$$

where ${\tilde{A}}^{o}={\left(\Gamma A\right)}^{*}⊙\left(\Gamma A\right)$.

Still utilizing the dictionary matrix described in Equation (10), the DOA estimation problem in the presence of miscalibrated sensors is formed as

(19) $$\underset{{\bar{p}}^{o}}{\min}\;{\left\|{\bar{p}}^{o}\right\|}_{0}\;s.t.\;{\left\|y^{o}-\bar{A}{\bar{p}}^{o}\right\|}_{2}^{2}\leq \varepsilon ,$$

which can also be relaxed to

(20) $$\underset{{\bar{p}}^{o}}{\min}\;\mu {\left\|{\bar{p}}^{o}\right\|}_{1}\;+\;{\left\|y^{o}-\bar{A}{\bar{p}}^{o}\right\|}_{2}^{2}.$$

According to Section 3.2, the MMSE criterion is non-optimal for this case.

Denote the residual errors between the reconstructed data and the measurements as

(21) $$e_{i}=\left|y_{i}^{o}-\sum_{d=1}^{D}{\bar{a}}_{i}\left(\theta_{d}\right){\bar{p}}_{d}^{o}\right|,$$

where $y_{i}^{o}$ and ${\bar{a}}_{i}\left(\theta_{d}\right)$ are the *i*-th element of $y^{o}$ and $\bar{a}\left(\theta_{d}\right)$ respectively, $i=1,2,\ldots ,{\left(2M+N-1\right)}^{2}$. The MCC for this case is then defined by

(22) $$\underset{{\bar{p}}^{o}}{\max}\;\frac{1}{{\left(2M+N-1\right)}^{2}}\sum_{i=1}^{{\left(2M+N-1\right)}^{2}}\kappa_{\sigma }\left(e_{i}\right).$$

Replacing the MMSE criterion by the MCC, the optimization problem in Equation (20) is reformed as

(23) $$\begin{array}{l} \underset{{\bar{p}}^{o}}{\max}\;-\mu {\left\|{\bar{p}}^{o}\right\|}_{1}+\frac{1}{{\left(2M+N-1\right)}^{2}}\;\sum_{i=1}^{{\left(2M+N-1\right)}^{2}}\kappa_{\sigma }\left(\left|y_{i}^{o}-\sum_{d=1}^{D}{\bar{a}}_{i}\left(\theta_{d}\right){\bar{p}}_{d}^{o}\right|\right)\\ \Leftrightarrow \underset{{\bar{p}}^{o}}{\max}\;-\mu {\left\|{\bar{p}}^{o}\right\|}_{1}+\frac{1}{{\left(2M+N-1\right)}^{2}}\;\sum_{i=1}^{{\left(2M+N-1\right)}^{2}}\kappa_{\sigma }\left(e_{i}\right) \end{array}.$$

The optimization problem in Equation (23) can be viewed as a dual objective optimization problem, in which the *l*_1_-norm term ensures the sparsity of ${\bar{p}}^{o}$ and the correntropy term ensures the estimation accuracy.

However, the objective function in Equation (23) is non-convex, which makes it difficult to solve. Fortunately, the half-quadratic (HQ) optimization technique can be applied to address this problem [26]. Based on the property of convex conjugate function [27], the following proposition is derived.

**Proposition** **1.** *There exists a convex conjugate function* Δ(ω) *of* $\kappa_{\sigma }\left(e\right)$ *, such that*

(24) $$\kappa_{\sigma }\left(e\right)=\underset{\omega }{\sup}\left(\omega \frac{{\left|e\right|}^{2}}{2\sigma^{2}}-\Delta (\omega )\right),$$

*and for a fixed e, the supremum is reached at $\omega =-\kappa_{\sigma }\left(e\right)$.*

With **Proposition 1,** the objective function in Equation (23) can be converted into an enlarged parameter space,

(25) $$\hat{F}({\bar{p}}^{o},\Omega )=-\mu {\left\|{\bar{p}}^{o}\right\|}_{1}+\frac{1}{{\left(2M+N-1\right)}^{2}}\;\sum_{i=1}^{{\left(2M+N-1\right)}^{2}}\left(\omega_{i}\frac{e_{i}^{2}}{2\sigma^{2}}-\Delta \left(\omega_{i}\right)\right)$$

where $\Omega ={\left[\omega_{1},\ldots ,\omega_{{\left(2M+N-1\right)}^{2}}\right]}^{T}$ is the vector of auxiliary variables. Denoting the original objective function in Equation (23) as $\hat{G}(z)$, according to **Proposition 1**, the following equation holds

(26) $$\hat{G}({\bar{p}}^{o})=\underset{\Omega }{\sup}\;\hat{F}({\bar{p}}^{o},\Omega ).$$

It holds that

(27) $$\underset{z}{\max}\;\hat{G}({\bar{p}}^{o})=\underset{z,\Omega }{\max}\;\hat{F}({\bar{p}}^{o},\Omega ),$$

from which, it can be concluded that maximizing the augmented function $\hat{F}(z,\Omega )$ on the enlarged parameter space is equivalent to maximizing $\hat{G}(z)$. Exploiting the strategy of alternate maximization, the value of augmented function in Equation (27) can be maximized in an iterative way. Suppose that the *q*-th iteration results have been obtained as ${\bar{p}}^{o}{}^{\left(q\right)}$ and $\Omega^{\left(q\right)}$, then the ${\bar{p}}^{o}{}^{\left(q+1\right)}$ and $\Omega^{\left(q+1\right)}$ can be calculated as follows

(28) $$\omega_{i}^{\left(q+1\right)}=-\kappa_{\sigma }(e_{i}^{\left(q\right)}),$$

(29) $$\begin{array}{ll} {\bar{p}}^{o}{}^{\left(q+1\right)} & =\arg\underset{{\bar{p}}^{o}}{\max}\;\frac{1}{{\left(2M+N-1\right)}^{2}}\;\sum_{i=1}^{{\left(2M+N-1\right)}^{2}}\left(\omega_{i}^{\left(q+1\right)}\frac{{\left|y_{i}^{o}-\sum_{d=1}^{D}{\bar{a}}_{i}\left(\theta_{d}\right){\bar{p}}_{d}^{o}\right|}^{2}}{2\sigma^{2}}-\Delta \left(\omega_{i}^{\left(q+1\right)}\right)\right)-\mu {\left\|{\bar{p}}^{o}\right\|}_{1}\\ & =\arg\underset{{\bar{p}}^{o}}{\max}\;\frac{1}{{\left(2M+N-1\right)}^{2}}\;\sum_{i=1}^{{\left(2M+N-1\right)}^{2}}\left(\omega_{i}^{\left(q+1\right)}\frac{e_{i}^{2}}{2\sigma^{2}}-\Delta \left(\omega_{i}^{\left(q+1\right)}\right)\right)-\mu {\left\|{\bar{p}}^{o}\right\|}_{1} \end{array}.$$

Since the conjugate function $\Delta (\omega_{i})$ is a function of auxiliary parameter $\omega_{i}$ and is independent of z at each iteration, the optimization problem Equation (29) can be simplified to

(30) $${\bar{p}}^{o}{}^{\left(q+1\right)}=\arg\underset{{\bar{p}}^{o}}{\max}\;\frac{1}{{\left(2M+N-1\right)}^{2}}\;\sum_{i=1}^{{\left(2M+N-1\right)}^{2}}\left(\omega_{i}^{\left(q+1\right)}\frac{e_{i}^{2}}{2\sigma^{2}}\right)-\mu {\left\|{\bar{p}}^{o}\right\|}_{1}.$$

Apparently, the objective function in Equation (30) is concave, so it can be efficiently solved using the interior point methods.

The relative error is used as convergence criterion. Specifically, the proposed method is said to be converged if the following inequality holds

(31) $$\left|\frac{{\hat{F}}^{\left(q\right)}\left({\bar{p}}^{o},\Omega \right)-{\hat{F}}^{\left(q-1\right)}\left({\bar{p}}^{o},\Omega \right)}{{\hat{F}}^{\left(q-1\right)}\left({\bar{p}}^{o},\Omega \right)}\right|\leq \varepsilon_{c},$$

where $\varepsilon_{c}$ is a user-specific tolerance parameter. In addition, the kernel size σ can be selected by Silverman’s rule at each iteration [25,28]:

(32) $$\sigma =1.06\times \min\left\{\sigma_{E},R/1.34\right\}\times M^{-0.8},$$

where $\sigma_{E}$ denotes the standard deviation of error $e_{i}$, and *R* denotes the error interquartile range.

### 4.2. Multiresolution Grid Refinement

One of the fundamental assumptions of the CS-based DOA estimation methods is that all the source locations are confined to a predefined grid. This grid is usually uniformly partitioned, and obviously a fine grid can ensure the estimation accuracy. However, the spatial domain cannot be uniformly partitioned into a overelaborate grid since it will dramatically increase the computational complexity. To reconcile the contradiction between estimation accuracy and computational complexity, an adaptive gird refinement strategy was developed in Ref. [29], and it is introduced into the proposed method. Instead of having a uniform grid, the grid is refined only around the potential during the iteration.

**Figure 2:**
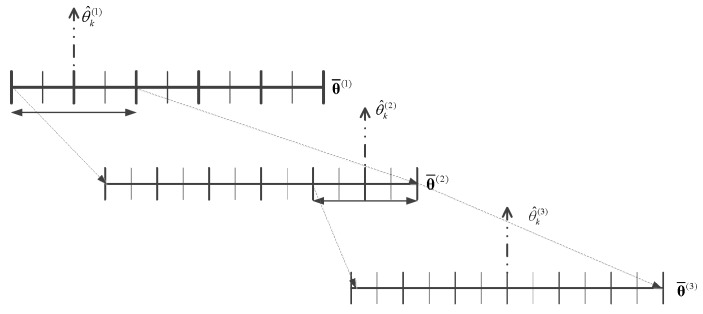
Illustration of grid refinement, where ${\hat{\theta }}_{k}^{\left(q\right)},k=1,\ldots K,q=1,\ldots ,Q$ is the estimated DOA of the *k*-th source at the *q*-th iteration, and ${\bar{\theta }}^{\left(q\right)}$ stands for the gird around ${\hat{\theta }}_{k}^{\left(q\right)}$ at the *q*-th iteration.

At the first iteration, the grid is initialized as a uniform coarse grid, and the spacing of the grid is denoted as $\delta^{\left(1\right)}$. It is worth noting that the initial grid should not be too rough to introduce substantial bias. The dictionary matrix ${\bar{A}}^{\left(1\right)}$ is generated by the coarse grid, and the optimization problem Equation (30) is then solved. Thus, the preliminary estimation of DOA is obtained as ${\hat{\theta }}_{k}^{\left(1\right)},\;k=1,\ldots ,K$. Pick up an interval around the *k*th estimated DOA, which includes two grid spacings to either side, i.e., $\left[{\hat{\theta }}_{k}^{\left(1\right)}-2\delta^{\left(1\right)},{\hat{\theta }}_{k}^{\left(1\right)}+2\delta^{\left(1\right)}\right]$, for k=1,…,K. New subgrids are created by repartitioning these picked intervals, whose spacing is a fraction of the old one, i.e., $\delta^{\left(2\right)}=\delta^{\left(1\right)}/\chi$. Here, χ is a predefined integer, and it is typically set to 3. The newly acquired grid sample points are added into the dictionary, and thus a new dictionary matrix ${\bar{A}}^{\left(2\right)}$ is formed for the next iteration. Note that the number of columns of the dictionary matrix ${\bar{A}}^{\left(2\right)}$ is increased after the grid refinement process, so the dimension of the vector ${\bar{p}}^{o\left(2\right)}$ should also be increased accordingly. The dimension expansion of ${\bar{p}}^{o\left(2\right)}$ can be accomplished by adding zero elements into it at the position corresponding to the newly acquired grid sample points. The expanded vector, referred to as ${\bar{p}}_{r}^{o\left(2\right)}$, is used as the initial value for the next iteration. Repeating the grid refinement process at each iteration, a non-uniform mesh is eventually generated. Figure 2 illustrates the details of the refinement procedure. An important issue in the grid refinement procedure is that the spacing of the subgrids cannot be infinitely small; otherwise the columns of the dictionary matrix become perfectly correlated [19]. So, the minimum spacing length $\delta_{m}$ is set, i.e., denoting the spacing of a locally uniform grid (piecewise uniform) at the *q*-th iteration as $\delta_{k}^{\left(q\right)}$; if $\delta_{k}^{\left(q\right)}\leq \delta_{m}$, the corresponding interval will not be further partitioned.

The proposed coprime array-based robust DOA estimation algorithm is now summarized and tabulated in Algorithm 1.

**Algorithm 1.** The proposed coprime array-based robust DOA estimation algorithm.**Input:** Coprime array received signals ${\left\{x(t)\right\}}_{t=1}^{T}$ **Output:** DOA Estimation ${\hat{\theta }}_{k},k=1,2,\ldots ,K$ **1.** Initialization: Set $\sigma^{\left(1\right)}=\infty$, ${\bar{p}}^{o(1)}=0$, maximum number of iteration *Q*, and index of iteration q=1; Initialize the dictionary matrix ${\bar{A}}^{\left(1\right)}$ with a coarse uniform grid. **2.** Calculate the covariance matrix ${\hat{R}}_{x}^{o}$ by Equation (5), and derive the second-order signal vector by Equation (18). **3.** Compute $\omega_{i}^{\left(q+1\right)}$ by equations Equation (21) and Equation (28). **4.** Optimize Equation (30) with $\omega_{i}^{\left(q+1\right)}$ to get the sparse vector ${\bar{p}}^{o(q+1)}$. **5.** Update the kernel size σ using Equation (32). **6.** Refine the grid; then reform the dictionary matrix to ${\bar{A}}^{\left(q+1\right)}$ and the sparse vector to ${\bar{p}}_{r}^{o(q+1)}$ according to the strategies given in Section 4.2. **7.** Set q←q+1. Repeat Step **2.** to Step **6.** until the convergence condition Equation (31) is reached or *q* exceeds *Q.*

The proposed algorithm has the following key advantages. First, by replacing the MMSE criterion with the MCC, the effect of the outliers is suppressed during the DOA estimation process and thus higher estimation accuracy can be achieved. Second, utilizing the property of the conjugate function, the proposed method is designed in an iterative form under the convex relax framework, which can be efficiently solved. Besides, the number of sources does not need to be known a priori. Third, employing the coprime array, the proposed robust DOA estimation method can make use of the enhanced DOF offered by the array, i.e., it can be used to find more sources than sensors. Last but not least, with the grid refinement strategy, the grid mismatch problem is alleviated in the proposed method.

### 4.3. Cramér-Rao Bound

In this subsection, the Cramér-Rao Bound (CRB), the lower bound of the minimum variance of an unbiased estimator, is derived for the coprime array in the presence of miscalibrated sensors. CRB is the inverse of the Fisher information matrix (denoted as **F**). Under the assumption that the gain and phase distortion parameters are unknown but deterministic, the (*i*, *j*)-th element of **F** can be represented as

(33) $${\left[F\right]}_{i,j}=T\mathrm{tr}\left[R_{x}^{-1}\frac{\partial R_{x}}{\partial \xi_{i}}R_{x}^{-1}\frac{\partial R_{x}}{\partial \xi_{j}}\right],$$

where $\xi_{i}$ and $\xi_{j}$ are the elements in the deterministic parameter vector ξ.

However, when the number of sources exceeds the number of sensors in the array, the **F** defined in Equation (33) becomes singular, which makes the CRB inapplicable. To address this problem, the vectorization process introduced in Ref. [30] is adopted, by which the **F** is reformed as

(34) $$F=T{\left[\mathrm{vec}\left(\frac{\partial R_{x}}{\partial \xi^{T}}\right)\right]}^{H}{\left(R_{x}^{T}⊗R_{x}\right)}^{-1}\left[\mathrm{vec}\left(\frac{\partial R_{x}}{\partial \xi^{T}}\right)\right].$$

In this form, the F keeps nonsingular within a much broader range of conditions.

In the case of this work, the deterministic parameter vector is defined by

(35) $$\xi ={\left[\theta^{T},\rho^{T},\phi^{T},p^{T},\sigma_{n}^{2}\right]}^{T},$$

where $\rho ={\left[\rho_{1},\ldots ,\rho_{2M+N-1}\right]}^{T}$ and $\phi =\left[\phi_{1},\ldots ,\phi_{2M+N-1}\right]$ denote the gain distortion parameter vector and the phase distortion parameter vector respectively. Let P denote diag(ρ) and Φ denote diag(ϕ), then the distortion parameter matrix can be expressed as Γ=PΦ. Additionally, denote the distorted steering matrix PΦA as $P\Phi A=H=[h_{1},\ldots ,h_{K}]$ (i.e., $h_{i}$ is the *i*-th column of H), and denote the vector ${\left[P\Phi A\right]}^{*}⊙\left[P\Phi A\right]p$ as ${\left[P\Phi A\right]}^{*}⊙\left[P\Phi A\right]p={\left[c_{1},\ldots ,c_{2M+N-1}\right]}^{T}$. Accordingly, the **F** can be specified as

(36) $$F=T{\left[\frac{\partial y^{o}}{\partial \xi^{T}}\right]}^{H}{\left(R_{x}^{T}⊗R_{x}\right)}^{-1}\left[\frac{\partial y^{o}}{\partial \xi^{T}}\right]$$

where

(37) $$\begin{array}{l} \frac{\partial y^{o}}{\partial \xi^{T}}=\left[\Lambda_{\theta }\vdots \Lambda_{\rho }\vdots \Lambda_{\phi }\vdots \Lambda_{p}\vdots \Lambda_{\sigma_{n}^{2}}\right],\\ \Lambda_{\theta }=\left[\frac{\partial y^{o}}{\partial \theta_{1}},\ldots ,\frac{\partial y^{o}}{\partial \theta_{K}}\right],\\ \Lambda_{p}=\left[\frac{\partial y^{o}}{\partial p_{1}},\ldots ,\frac{\partial y^{o}}{\partial p_{K}}\right],\\ \Lambda_{\sigma_{n}^{2}}=\left[\frac{\partial y^{o}}{\partial p_{\sigma_{n}^{2}}}\right] \end{array}$$

with

(38) $$\begin{array}{l} \Lambda_{\rho }={\left[{\left[\frac{\partial c_{1}}{\partial \rho^{T}}\right]}^{T},\ldots ,{\left[\frac{\partial c_{{\left(2M+N-1\right)}^{2}}}{\partial \rho^{T}}\right]}^{T}\right]}^{T},\\ \Lambda_{\phi }={\left[{\left[\frac{\partial c_{1}}{\partial \phi^{T}}\right]}^{T},\ldots ,{\left[\frac{\partial c_{{\left(2M+N-1\right)}^{2}}}{\partial \phi^{T}}\right]}^{T}\right]}^{T},\\ \frac{\partial y^{o}}{\partial \theta_{k}}=p_{k}\left[\frac{\partial h_{k}^{*}}{\partial \theta_{k}}⊗h_{k}+h_{k}^{*}⊗\frac{\partial h_{k}}{\partial \theta_{k}}\right],\\ \frac{\partial y^{o}}{\partial p_{k}}=h_{k}^{*}⊗h_{k},\\ \frac{\partial y^{o}}{\partial \sigma_{n}^{2}}=i \end{array}$$

Therefore, the CRB for the *k*-th source can be obtained as

(39) $$\mathrm{CRB}\left(\theta_{k}\right)={\left[F^{-1}\right]}_{k,k},$$

for 1≤k≤K.

## 5. Simulation

In this section, a series of numerical simulations are conducted to examine the performance of the proposed method. In these simulations, the pair of coprime integers is chosen as M=3,N=5 to deploy the extended coprime array. There are 2M+N−1=10 sensors in the array, which are located at {0,3d,5d,6d,9d,10d,12d,15d,20d,25d}. The proposed robust DOA estimation algorithm is compared to several recently-reported DOA estimation algorithms utilizing the coprime array, namely the Spatial Smooth MUSIC algorithm (SS-MUSIC) [4], the Nuclear Norm Minimization (NNM) algorithm [31], and the Sparse Signal Reconstruction (SSR) algorithm [7]. Two CS-based self-calibration methods that do not require prior knowledge of the number of sources are also compared, namely the Sparse-Based Array Calibration algorithm (SBAC algorithm in Ref. [17]) and the Sparse Bayesian learning Array Calibration algorithm (SBAC algorithm in Ref. [21]). The sampling interval of the predefined grid is set as $0.1^{\circ }$ for the SSR algorithm, the SBAC algorithm in Ref. [17] and the SBAC algorithm in Ref. [21]. The regularization parameter τ and precision parameter ε for the SBAC algorithm in Ref. [17] are set as 0.1 and 0.01 respectively. For the proposed algorithm, the sampling interval of the initial coarse grid is selected to be $1^{\circ }$, and the minimum spacing length $\delta_{m}$ is selected to be $0.05^{\circ }$.The regularization parameter μ for the SSR algorithm and the proposed algorithm is empirically set to be 0.25. The tolerance parameter $\varepsilon_{t}$ and the maximum number of iterations *Q* for the proposed algorithm are set as $10^{-5}$ and 50 respectively. The convex optimization problems are solved by using the CVX [32].

**Figure 3:**
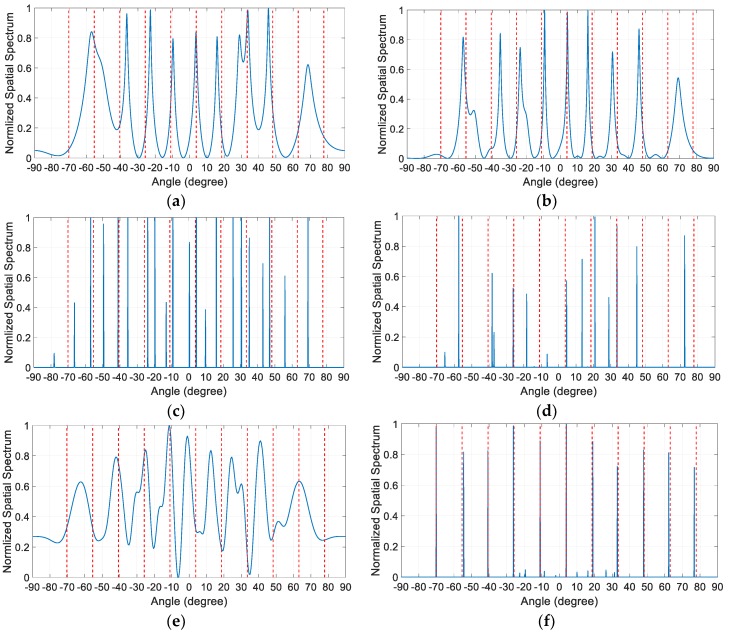
Normalized spatial spectrum comparison with SNR = 30dB and the number of snapshots. *T* = 2000 when *K* = 11 and M={4,6,8}. (**a**) SS-MUSIC algorithm; (**b**) NNM algorithm; (**c**) SSR algorithm; (**d**) SBAC algorithm in Ref. [17]; (**e**) SBAC algorithm in Ref. [21]; (**f**) Proposed algorithm.

For the first three examples, it is assumed that the *K* = 11 equal-power sources uniformly distributed in [−70.25,77.85] impinge on the array. In the first example, the spatial spectra estimated by these algorithms in the presence of miscalibrated sensors are compared. The sensor miscalibrations are assumed to occur on the fourth, sixth and eighth sensors in the array, i.e., M={4,6,8}. The specific values of the distortion parameters of the miscalibrated sensors are 3exp(j4), 3exp(j2) and 4exp(j4) respectively. It is worth mentioning that both the locations and the distortion parameters of the miscalibrated sensors are unknown a priori. The SNR of all the sources is set to be 30 dB, and *T* = 2000 snapshots of the received signals are collected for DOA estimation. The normalized spatial spectra are depicted in Figure 3, where the vertical dashed lines denote the actual directions of the incident sources.

It is observed from Figure 3a,b that the number of obvious sharp peaks in the spatial spectra of SS-MUSIC and NNM algorithm is less than the source number *K*, which makes it hard to correctly identify the number of sources. Moreover, most of the estimation results of these two algorithms deviate from the actual source directions. As shown in Figure 3c, the SSR algorithm cannot correctly identify the number of sources either, and only a few of the estimation results of it are close to the actual source directions. However, unlike the SS-MUSIC and NNM algorithm, the number of peaks in the spatial spectrum of SSR algorithm is more than the sources number. Figure 3d,e show that neither the SBAC algorithm in Ref. [17] nor the SBAC algorithm in Ref. [21] can properly calibrate the gain and phase distortions under such serious disturbance. Most of the estimation results of them still deviate from the actual source directions. In contrast, the proposed algorithm is able to correctly resolve all the peaks in the actual source direction.

**Figure 4:**
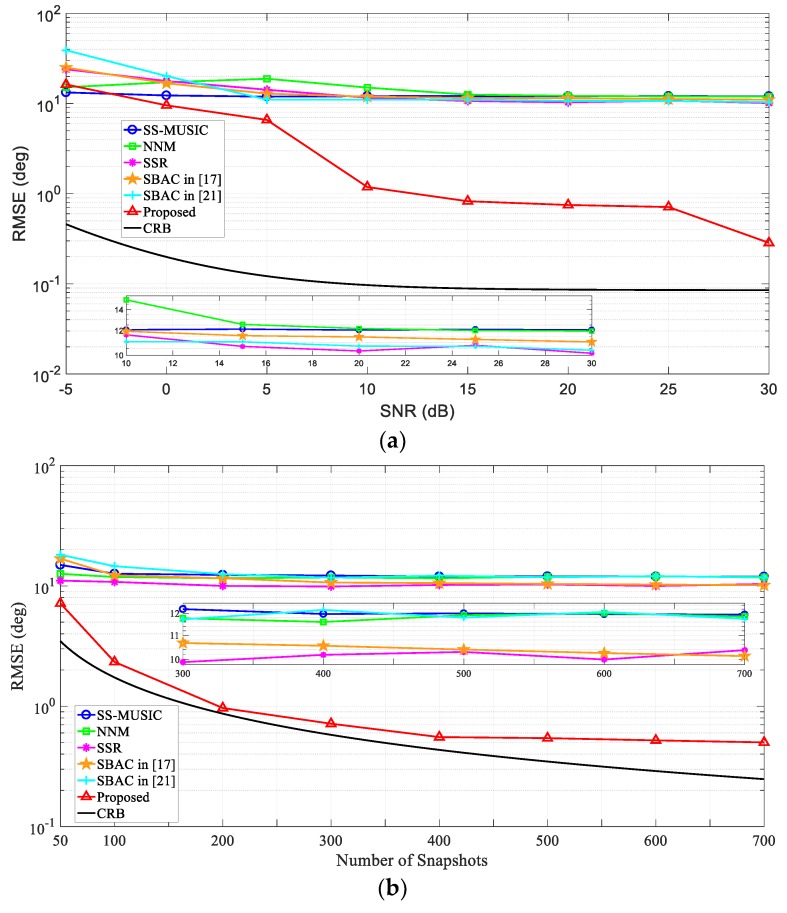
RMSE performance comparison with 11 incident sources. (**a**) RMSE versus SNR with the number of snapshots *T* = 2000; (**b**) RMSE versus the number of snapshots with SNR = 30 dB.

In the second example, the root mean square error (RMSE) of each algorithm is compared in Figure 4. Here, the RMSE is defined as

(40) $$\mathrm{RMSE}=\sqrt{\frac{1}{KQ_{M}}\sum_{k=1}^{K}\sum_{q_{M}=1}^{Q_{M}}\left({\hat{\theta }}_{k}\left(q_{M}\right)-\theta_{k}\right)},$$

where ${\hat{\theta }}_{k}\left(q_{M}\right)$ is the estimated DOA of the *k*-th source in the $q_{M}-\mathrm{th}$ Monte Carlo trial, and $Q_{M}$ is the number of Monte Carlo trials. It can be seen from example 1 that, under some conditions, some of the algorithms cannot correctly distinguish the number of sources. Therefore, for statistical convenience, the number of sources, *K*, is considered as known, and the angels corresponding to *K* highest peaks in spatial spectra are picked as estimated DOAs. The number of snapshots is fixed at *T* = 2000 when the SNR varies, whereas the SNR is fixed at 30 dB when the number of snapshots varies. The locations and the distortion parameters of the miscalibrated sensors are set to the same as in example 1. For each data point, $Q_{M}=500$ Monte Carlo trials are conducted to calculate the RMSE. The Cramér-Rao bound (CRB) Equation (39) is also plotted.

It can be seen from Figure 4a that, when there are some miscalibrated sensors in the array, the estimation accuracy of SS-MUSIC algorithm, NNM algorithm and SSR algorithm cannot be significantly improved by increasing SNR. Compared with these three uncalibrated methods, the RMSE performances of two self-calibration methods show no significant improvement. As for the proposed algorithm, it does not show superiority in estimation accuracy when SNR≤0 dB. However, when SNR is lager 5 dB, the proposed algorithm obviously outperforms other algorithms in terms of RMSE. The reason lies in that when SNR is low (lower than 0 dB), the outliers cannot be effectively distinguished by using correntropy. As shown in Figure 4b, increasing the number of snapshots is not helpful to improve the estimation accuracy of the SS-MUSIC algorithm, NNM algorithm and SSR algorithm, either. Meanwhile, neither the SBAC algorithm in Ref. [17] nor the SBAC algorithm in Ref. [21] can improve estimation accuracy. The proposed algorithm outperforms other algorithms even when the number of snapshots is small. Comparing Figure 4a with Figure 4b, it can be also found that the proposed MCC-based algorithm is more sensitive to SNR than to the number of snapshots. The RMSE of the proposed algorithm still decreases significantly when the SNR increases from 25 dB to 30 dB.

In the third example, both the DOA estimation accuracy and the robustness of the tested algorithms are compared in the presence of miscalibrated sensors. First, the deviation distance of estimation (DDOE) of the $q_{M}-\mathrm{th}$ Monte Carlo trial is defined as

(41) $${\mathrm{DDOE}}^{\left(q_{M}\right)}=\sqrt{\sum_{k=1}^{K}\left({\hat{\theta }}_{k}(q_{M})-\theta_{k}\right)}.$$

**Figure 5:**
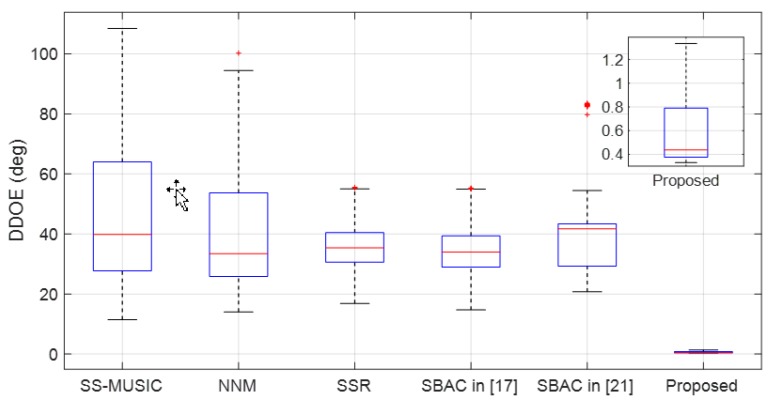
Box plots of DDOE for the tested algorithms.

For the same reason as stated in example 2, the number of sources, *K*, is considered as known. The SNR and the number of snapshots in this example are set as 30 dB and 2000 respectively. It is assumed that there are three miscalibrated sensors in the array, whose locations are randomly selected from S in each Monte Carlo trial. The gain distortion parameter $\rho_{m}$ of each miscalibrated sensor is randomly selected from the interval [3,5], and the phase distortion parameter $\phi_{m}$ of each miscalibrated sensor is randomly selected from the interval [2,4] in each Monte Carlo trial. Figure 5 gives the box plots of DDOE of the tested algorithms, where the statistical data is collected from 500 Monte Carlo trials for each algorithm.

Figure 5 shows that variation range of DDOE of the CS-based DOA estimation algorithms are smaller than the other two algorithms. However, neither the SBAC algorithm in Ref. [17] nor the SBAC algorithm in Ref. [21] show higher robustness or better estimation accuracy than the SSR algorithm. Moreover, the proposed algorithm shows the best robustness with the minimum box height and the best estimation accuracy with smallest mean value.

**Figure 6:**
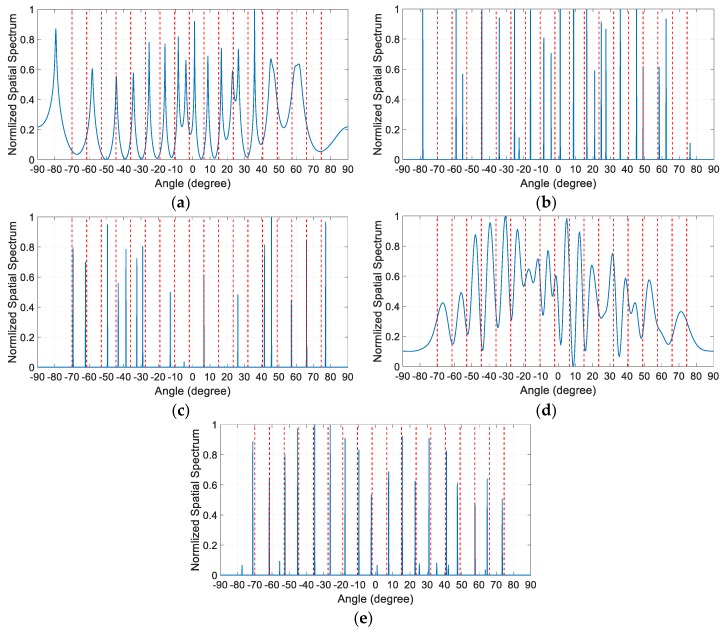
Normalized spatial spectrum comparison with SNR = 30 dB and the number of snapshots *T* = 2000 when *K* = 18 and M={4,8}. (**a**) NNM algorithm; (**b**) SSR algorithm; (**c**) SBAC algorithm in Ref. [17]; (**d**) SBAC algorithm in Ref. [21]; (**e**) Proposed algorithm.

In the fourth example, it is assumed that *K* = 18 equal-power sources uniformly distributed in $\left[-70^{\circ },74.5^{\circ }\right]$ impinge on the array. The sensor miscalibration is assumed to occur on the fourth and eighth sensors in the array, i.e., M={4,8}. The specific values of the distortion parameters of the miscalibrated sensors are 3exp(j2) and 2exp(j3) respectively. In this case, the number of sources is equal to the number of virtual sensors in the contiguous part of the difference coarray; therefore, the SS-MUSIC algorithm cannot be used. The normalized spatial spectra of the remaining three algorithms are compared in Figure 6 with the SNR = 30 dB and the number of snapshots *T* = 2000, where the vertical dashed lines also denote the actual directions of the incident sources. The comparison results shown in Figure 6 are similar to those in Example 1, which also verifies the superiority of the proposed algorithm.

## 6. Conclusions

In this paper, a novel robust DOA estimation algorithm for coprime array in the presence of miscalibrated sensors has been proposed. The proposed method employs the compressive sensing (CS)-based algorithm and fully utilizes all the received signals, thus exploiting the enhanced DOF offered by the coprime array. Without the requirement for prior information of the miscalibrated sensors, the information received by them is blindly treated as outliers. The maximum correntropy criterion (MCC) is introduced into the sparse signal recovery process to suppress the influence the outliers, and then the process is constructed as an optimization problem. For the convenience of solving the constructed optimization problem, an iterative algorithm is developed by exploiting the property of conjugate function under the convex relax framework. Moreover, to alleviate the grid mismatch problem, the multiresolution grid refinement strategy is incorporated, with which the sampling grid is refined at each iteration. As a result, the proposed algorithm can find more sources than sensors with high DOA estimation accuracy when some sensors in the array are miscalibrated. Simulation results verified the robustness and the effectiveness of the proposed algorithm.
